# The struggle for existence in the world market ecosystem

**DOI:** 10.1371/journal.pone.0203915

**Published:** 2018-10-03

**Authors:** Viviana Viña-Cervantes, Michele Coscia, Renaud Lambiotte

**Affiliations:** 1 Naxys Department of Mathematics, University of Namur, Namur, Belgium; 2 Center for International Development, Harvard University, Cambridge, MA, United States of America; 3 IT University of Copenhagen, Copenhagen, Denmark; 4 Mathematical Institute, University of Oxford, Oxford, United Kingdom; Northeastern University, CHINA

## Abstract

The global trade system can be viewed as a dynamic ecosystem in which exporters struggle for resources: the markets in which they export. We can think that the aim of an exporter is to gain the entirety of a market share (say, car imports from the United States). This is similar to the objective of an organism in its attempt to monopolize a given subset of resources in an ecosystem. In this paper, we adopt a multilayer network approach to describe this struggle. We use longitudinal, multiplex data on trade relations, spanning several decades. We connect two countries with a directed link if the source country’s appearance in a market correlates with the target country’s disappearing, where a market is defined as a country-product combination in a given decade. Each market is a layer in the network. We show that, by analyzing the countries’ network roles in each layer, we are able to classify them as out-competing, transitioning or displaced. This classification is a meaningful one: when testing the future export patterns of these countries, we show that out-competing countries have distinctly stronger growth rates than the other two classes.

## Introduction

Global trade can be considered as a complex system, whose sophisticated behavior emerges from its many interacting parts—countries exporting products in different importing markets. This systemic view has been adopted in the past and it proved to be an effective one. Diversity and product relatedness in the export basket of countries and regions has been used as proxy of their economic solidity [1–4]. Different economic complexity indexes have proven to be incredibly successful in predicting future economic growth, better than traditional indicators such as years of schooling or the quality of public institutions (e.g. in terms of resistance to corruption) [5–8]. The complexity approach illustrated how knowledge flows across neighboring countries [9, 10], and how these dynamics allow us to predict structural change [11, 12], suggesting new avenues for development [13].

Here, we enrich the literature on complexity and economic development by further investigating its relationship with ecology. Traditionally, export patterns are considered as static and only locally related to the other countries in the world. The classical first step is to calculate the Revealed Comparative Advantage [14] of a country in a given product across all importers for a given time interval. Instead, we draw relations among countries by inferring potential competition among them across time. We see a pair of importer-product, for instance the car market in the US, as an evolving trade “niche”, with exporters appearing and disappearing like fit and unfit organisms in an ecosystem. In our analysis, the fitness of an economy in a niche correlates with its ability to displace (out-compete) unfit economies. If this happens consistently in many other car importing countries, then the fit economy should be able to grow its car exporting business in the future.

**Fig 1:**
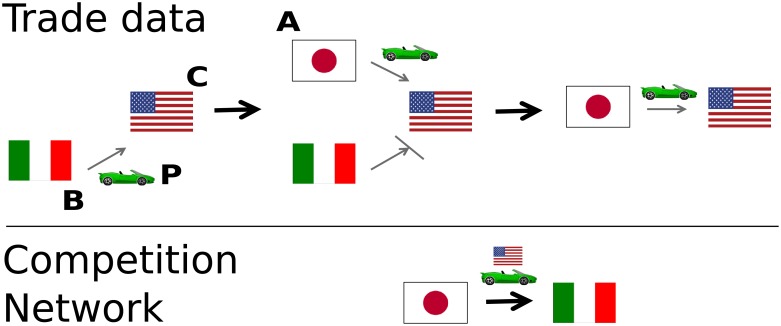
An example of a displacement relation. From left to right we observe a pattern in the yearly car import data from the US. In the first year, only Italy is present. In the second year, Japan appears in the market. In the third year, Italy disappears from the market. This pattern from the trade data is represented in the competition network as a directed edge from the displacer (Japan) to the displaced (Italy). The edge is labeled with its layer: the car market in the United States.

We test this theory by creating a competition network, connecting country *a* to country *b* if *a*’s appearance in a market preceded *b*’s disappearance, as illustrated in Fig 1. Since we have different products and different years in which these relationships can be established, we use a multilayer network model [15, 16]. Our competition network is a peculiar structure, because traditionally networks are used to express positive relations, while in our case the relation is negative (competition). Negative relationships are relatively less explored than positive ones, and previous works showed they obey to different dynamics [17, 18], whether they are studied using social balance or status theory frameworks [19, 20]. For instance, negative edges are much less prone to generate triangles and high clustering [21, 22]. They also allow for the emergence of more complex network motifs [23].

World’s markets are highly dynamic, with exporters frequently appearing and disappearing in a niche. This means that basic statistical properties of our competition network are not enough to unveil the potential displacement patterns. In the competition network there is a very strong correlation between out- and in-degree, which record the number of displacements a country caused and to which it was subject, respectively. As a consequence, it is not possible to detect if a country tends to out-compete more than it is out-competed. Moreover, in the competition network we observe a number of unexpected properties, such as reciprocity—countries repeatedly displacing each other—and triangles—cycles of countries where the displacers are displaced by their displaced’s displaced. To tackle these issues, we need to employ non-local analysis techniques, and take into account indirect patterns in the directed graph. This is an approach frequently used in network science, from ranking a node’s structural importance [24], to the measurement of node similarity [25].

In this paper, we choose to borrow the tools of a third non-local node-centric network analysis: role detection. In the role detection literature, different connectivity patterns are used to classify nodes in particular network roles [26]. One specific and very popular case is the one of community detection, which aims at finding densely connected modules [27]. Node roles have been used to describe a wide range of phenomena, from metabolic networks [28] to the connectivity in the brain [29, 30]. Specifically, we borrow the approach described in [31]. In this method, we compute a feature vector for each node describing the size of the out/in neighborhoods at a given network distance. Through this vector, we redefine a fit economy from “able to out-compete many countries” to “able to out-compete many countries who are able to out-compete many countries”—up to six degrees of separation. We perform the same operation for in-degree roles (an unfit exporter is an exporter who is “displaced by countries who are prone to be displaced themselves”).

We define three roles for exporters: “Out-competing” countries are countries which consistently score high in out-degree roles and low in in-degree roles; “Displaced” countries are countries scoring the opposite (low in out-degree roles and high in in-degree roles); and “Transitioning” countries, whose scores in both roles are comparable.

This classification is a meaningful one. We test it by predicting the future export patterns of countries. Countries classified as out-competing in a particular product in a particular decade show significant export growth patterns in that product in the following decade. This means, for instance, that if Japan is classified as out-competing in worldwide car exports in the 1960-1970 decade, then its car exports are going to grow significantly in the 1970-1980 decade. This result is consistent across decades—with the exception of the last decade for the lack of a long enough time span to test the data—and across different product types—with the exception of the ones dominated by profitable natural resources such as crude oil.

Even if we are not observing direct competition relationships, due to the correlative nature of our edge creation process, the resulting roles are informative of future patterns in global trade. Our method can be used to detect emerging countries in the global market for a particular product.

## Methods

The aim of this section is to describe the process starting from raw trade data to the creation and analysis of multilayer competition network. We start by describing the data sources and the cleaning phase. We then provide an informal example, before detailing out the full procedure.

### Data & cleaning

The data contains the entire set of worldwide trade relationships from 1962 until 2013. The data has been collected by the UN Comtrade organization (https://comtrade.un.org/), and cleaned by CEPII [32]. A product is defined as a 4 digit SITC category. A product can be, for instance, poultry meat for eating (code 0123), or ferro-manganese (code 6714).

UN Comtrade gathers data about all sovereign countries and territories in the world. Many of these sovereign entities are very small and cause wide fluctuations in the observations. For this reason, we focus only on larger and more stable countries. We drop countries with less than 300k inhabitants and/or with a total GDP lower than 300 million US dollars. Given our large time span, we also have data about countries who do not exist any more (for instance, Yugoslavia). We drop the observations involving them too.

Even if the data is gathered at a 4-digit level of detail, we find that this is too granular for our analytic aims. We exploit the fact that SITC is a hierarchical classification: all products whose code starts with the same digit are related to each other. Thus we aggregate the trade data at the 1-digit level, summing up the trade flows of all products classified under the first digit.

Finally, we represent the data as a four dimensional tensor $T_{p,i,e,y}$. The dimensions of the tensor are: product (*p*), importer (*i*), exporter (*e*) and year (*y*). Basically, $T_{p,i,e,y}$ can be seen as a set of matrices $T_{e,y}^{p,i}$, one for each pair of product *p* and importer *i*. The matrix contains, for each exporter *e* a timeline vector recording, for each year *y*, the amount of trade in *p* flowing from *e* to *i*. So, each $T_{e,y}^{p,i}$ is a *e* × *y* matrix.

### Inferring competition relationships

**Fig 2:**
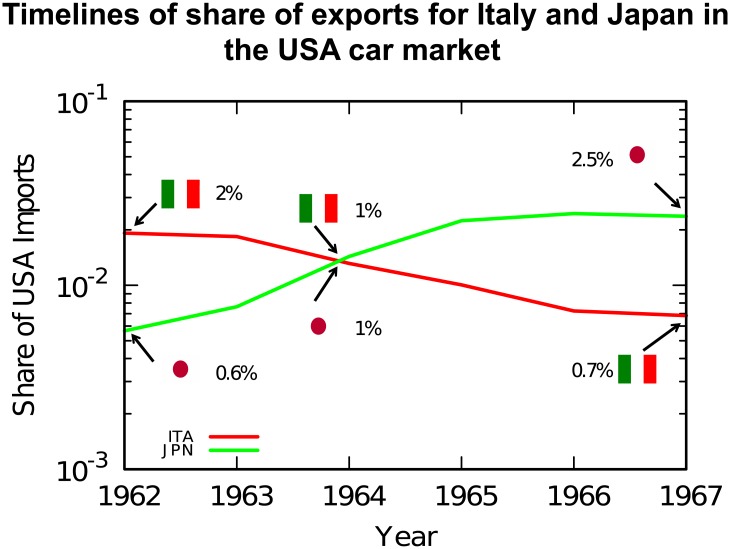
Timeline of Japan’s and Italy’s exports in the US car market in the 60s.

To better understand how the procedure works, let us start with an example detailing how a single edge in our multilayer directed network is established. We consider the car market in the United States. We focus on the export patterns of two countries in a potential relationship of competition: Japan and Italy. Fig 2 depicts the share of US car market of Japan and Italy, from 1962 to 1967.

Each step corresponds to a parameter in our methodology, which is reported between parenthesis, and which we formally define in the rest of this section:

1. Detect whether there is an anti-correlation between the export patterns of the two countries (*δ*);
2. Detect whether one of the two countries appeared from the market, while the other disappeared (*κ*);
3. Detect whether the disappearing country did not reappear in the market immediately after the event (λ).

In the first step we calculate the correlation coefficients of Japan’s and Italy’s export timelines. The two timelines have a 1.67 correlation distance. If we assume that this is higher than our *δ* parameter, we can say that there is a potential competition edge between Japan and Italy.

In the second step, we check if either country appeared in the market while the other disappeared. This is regulated by the parameter *κ*, which defines the relative market share below which an exporter is considered to have “disappeared”. We do not set *κ* = 0, because a complete disappearance is a rare event. If we set *κ* = 1%, we can say that Italy disappeared while Japan appeared, suggesting that the competition edge runs from Japan to Italy.

Finally, we check if Italy was absent from the US car market for at least λ years. Assuming λ = 2, also this final test is positive. We then draw a directed edge in our competition network from Japan to Italy.

We now describe more formally each step in the following subsections. We remind that the same operation is performed for each product 1-digit class (from 1 to 8 excluding 9, since it contains miscellaneous products not related to each other), and for each decade separately (1960-70, 1970-80, 1980-90, 1990-2000).

#### Step #1: Detecting the potential edges

To detect the candidate relationships (i.e. the edges), we slice $T_{p,i,e,y}$ such that we consider each pair of product and importer country independently from all other pairs, i.e. we analyze one $T_{e,y}^{p,i}$ matrix at a time. We column normalize each $T_{e,y}^{p,i}$, such that each entry will report the share *e* exported of *p* to *i* in *y*. We then calculate the row-wise correlation distance between each pair of exporting countries:

$$\begin{array}{c} d_{e_{1},e_{2}}=1-corr\left(\vec{e_{1}},\vec{e_{2}}\right), \end{array}$$

where *e*_1_ and *e*_2_ are two exporting countries, $\vec{e_{1}}$ and $\vec{e_{2}}$ are the vectors of $T_{e,y}^{p,i}$ corresponding to them, and *corr* is a function calculating the Pearson correlation of two vectors. $d_{e_{1},e_{2}}$ establishes the distance in the trends of *e*_1_ and *e*_2_, regardless of their relative volume. Remember that here we are interested in linking countries that are *dissimilar* to each other, so we perform an operation that is opposite to what is usually done in network science: two countries with very different market shares are not connected with an edge if their trends are similar.

$d_{e_{1},e_{2}}$ takes values between 0 ($\vec{e_{1}}$ and $\vec{e_{2}}$ are perfectly correlated) and 2 ($\vec{e_{1}}$ and $\vec{e_{2}}$ are perfectly anti-correlated). $d_{e_{1},e_{2}}$ equals to 1 for linearly uncorrelated vectors. The *δ* threshold establishes the value below which we discard the potential edge. Given the value domain of $d_{e_{1},e_{2}}$, *δ* must be higher than 1 (otherwise we would consider positively correlated vectors).

#### Step #2: Detecting the potential edge direction

To establish if the anti-correlation of exports can lead to a potential competition edge—and its direction—we have several requirements to satisfy:

1. *i* must have not stopped importing *p*;
2. Either *e*_1_ or *e*_2_ has to have ceased to export *p* to *i*—this is the potential displaced exporter;
3. Whenever *e*_1_ ceased to export *p* to *i*, *e*_2_ still has to be exporting the product, and vice versa—this is the potential out-competitor exporter;
4. The potential displaced exporter must have been exporting *p* previously.

To satisfy requirements #1, #2 and #3, we use our second threshold, *κ*, which represents the minimum export share to be considered still exporting *p* to *i*. If an exporter *e* has less than *κ* market share of *p* in *i*, then *e* in this context is considered to have ceased exporting. Being *κ* a relative threshold, we can make sure that the size of the importing market is not affecting our definition of relationship, which would make too easy to have competition relationships in small countries and small products.

Requirement #1 is now satisfied automatically: it is impossible to have a share of export larger than *κ* if the denominator is 0 (i.e. *i* did not import *p*), because the fraction would be undefined.

Each candidate edge is a quadruple (*p*, *i*, *e*_1_, *e*_2_). For each *p* and *i*, we binarize $\vec{e_{1}}$ and $\vec{e_{2}}$ as follows:

$$\begin{array}{c} e_{y}^{*}=\{\begin{array}{cc} 1 & \text{if}\;e_{y}>\kappa \\ 0 & \text{otherwise.} \end{array} \end{array}$$

where *e*_*y*_ is $\vec{e}$’s value at time *y*. Then we calculate ${\vec{e_{1}}}^{*}⊕{\vec{e_{2}}}^{*}$, which is the XOR product of the two vectors: the result is true for a year *y* if in *y* *e*_1_ exported more than *κ* share of *p* to *i* and *e*_2_ did not, and vice versa. This satisfies requirements #2 and #3.

We satisfy requirement #4 by removing the first streak of true values in ${\vec{e_{1}}}^{*}⊕{\vec{e_{2}}}^{*}$. The first streak of true values represents a period in which either *e*_1_ or *e*_2_ did not start exporting *p* to *i* yet. Thus, we cannot talk about either of them being displaced, because they did not have a chance to interact with each other yet.

We can now easily detect the edge direction. The country which disappeared from the importing market—say *e*_2_—is the displaced one and it is thus on the receiving end of the edge, which originates from the other country—in our case *e*_1_.

#### Step #3: Establishing the edge

Before adding the edge to the multilayer competition network we have to ensure that the displaced exporter has actually been displaced. We test this by checking if the cessation of its exports has been longer than a certain number of years.

We satisfy this requirement by using our third parameter, λ, which represents the minimum number of years needed to declare a potential displaced exporter out of the market. This means that the displaced country has to cease exporting at least *κ* share of *p* to *i* for λ consecutive years, while its out-competitor consistently stays above the *κ* threshold in the same period. This means that we have to find at least λ consecutive true values in ${\vec{e_{1}}}^{*}⊕{\vec{e_{2}}}^{*}$.

The result of these three steps is another tensor, $D_{p,d,e_{1},e_{2}}$. $D_{p,d,e_{1},e_{2}}$ is a directed multilayer network, where each layer represents a pair of product *p* and decade *d*. For simplicity, D is collapsed over the importer dimension *i* using a logical OR operator. In other words, each layer contains an directed graph connecting two countries (*e*_1_ → *e*_2_) if their trends in exporting *p* during *d* satisfy all posited requirements for at least one importer *i*:

- $\vec{e_{1}}$ and $\vec{e_{2}}$ are strongly anti-correlated (correlation distance > *δ*);
- $\vec{e_{2}}$ contains at least λ consecutive values < *κ* not at its beginning;
- The corresponding $\vec{e_{1}}$ values are ≥ *κ*.

We then say that *e*_1_ is an out-competitor of *e*_2_ in product *p*. The edges are weighted according to in how many importers *i* this competition relationship has been established.

### Detecting roles

We now turn to the detection of node roles in the multilayer competition network. We follow closely the methodology delineated in [31]. In that paper, Cooper and Brahona propose to group nodes according to their role in the network, defined in terms of the overall pattern of incoming and outgoing flows. According to this, we expect to find three categories of countries: out-performing, displaced and transitioning. The roles emerge by looking at the path profile of each node. A path profile is a vector computed from the powers of the adjacency matrix weighted with a scale parameter. Then, we define path profile templates and we cluster nodes according to the similarity their path profiles have when compared to the templates.

Consider a directed network with *N* nodes and an asymmetric adjacency matrix *M*. Consider its [*M*^*k*^ **1**] vector, where **1** is the *N* × 1 vector of ones. The i-th entry of this vector is the number of displacement events happening in all chains of length *k* originating from node *i*. For *k* = 1, [*M*^*k*^ **1**] is equivalent to the out-degree vector of *M*. In the same way, the number of displacement events happening in all chains of length *k* ending in node *i* is [*M*′^*k*^ **1**]_*i*_, where *M*′ is the transpose of *M*. For *k* = 1, this is equivalent to the in-degree vector of *M*.

We construct a matrix that compiles the incoming and outgoing paths of all lengths up to *k*_*max*_ by appending the column vectors indexed by path length and scaled by the factors *βk*:

$$\begin{array}{c} X=[\begin{array}{c} x_{1}\\ .\\ .\\ .\\ x_{N} \end{array}]\equiv \left[\underset{k_{max}}{\underset{︸}{\ldots {\left(\beta M^{\prime }\right)}^{k}1\ldots }}|\underset{k_{max}}{\underset{︸}{\ldots {\left(\beta M\right)}^{k}1\ldots }}\right], \end{array}$$

where *β* = *α*/λ_1_, with λ_1_ being the largest eigenvalue of the adjacency matrix and *α* > 0. *α* governs how much weight we put on local or global flow structure. Setting *α* ∼ 0 means that in- and out-degrees dominate over the other values when calculating roles. Given the issues caused by using in- and out-degree that we will describe in the next section, we aim at doing the exact opposite, and thus we set *α* = 1. Note that one could set an *α* > 1, however that would mean that the farther relationships (mediated by more than one edge) have more weight than the more proximate ones, which we believe not to be reasonable. We also consider up to 6 degrees of separation in each direction, i.e. *k*_*max*_ = 6.

By following this methodology, each row vector of *X* contains the flow profile of a node in terms of the scaled number of displacement paths of all lengths starting and ending at that node. Following [31], we group nodes if they have similar flow profiles. Nodes in the same cluster have similar flow profiles, thus they play a similar role in terms of the flow in the directed graph. To detect such nodes, we calculate the distance of each country from a synthetic template of a perfect out-competing, transitioning, and displaced exporter. We assign the country to the closest template according to the cosine distance. The objective is to minimize the average cosine distance between a country and its template.

To create our templates we need to ensure that each element in each row vector in *X* takes value between 0 and 1:

$$\begin{array}{c} X^{*}=\frac{X-min(X)}{max(X)-min(X)}. \end{array}$$

Note that this operation is done row by row, i.e. *min*(*X*) and *max*(*X*) are calculated only considering the values of each row separately. In this way, each country is a vector of values between 0 and 1 included. If we would took the global *min*(*X*) and *max*(*X*), only one country could span the full domain value, narrowing down the values of all other countries, and thus making the result dominated by outliers. As a result of this operation, a hypothetical country *i* could be described by the following vector:

$$\begin{array}{c} X_{i}^{*}=\left[\underset{d}{\underset{︸}{0.97,0.94,1,0.99,0.92,0.77}},\underset{o}{\underset{︸}{0.42,0.11,0.14,0.13,0,0.09}}\right]. \end{array}$$

Here, the first *k*_*max*_ values are the displaced (*d*) role scores, while the latter *k*_*max*_ values are the out-competing (*o*) role scores. As a convention, we always list first the *d* scores in decreasing order and then the *o* scores in increasing order, so that the two middle values of the vector are always *d*_1_ and *o*_1_, i.e. the normalized in-degree and the out-degree. Generally speaking, the *d*_*n*_ entry in the *i*th row of matrix X* is the (normalized) number of paths of length *n* ending at node *i*. A high score in displaced roles means that the country tends to be displaced by countries that are displaced themselves. The opposite is true for the out-competing role scores. Since we know that all scores must take value between 0 and 1, creating a cluster template is now trivial:

$$\begin{array}{c} O=[\underset{d}{\underset{︸}{0,0,0,0,0,0}},\underset{o}{\underset{︸}{1,1,1,1,1,1}}] \end{array}$$

is an hypothetically perfect out-competing exporter, with zero in-degree and maximum out-degree. With the same logic, we can define the perfect displaced country D, and the middle point, the transitioning country T:

$$\begin{array}{c} D=[\underset{d}{\underset{︸}{1,1,1,1,1,1}},\underset{o}{\underset{︸}{0,0,0,0,0,0}}], \end{array}$$

$$\begin{array}{c} T=[\underset{d}{\underset{︸}{0.5,0.5,0.5,0.5,0.5,0.5}},\underset{o}{\underset{︸}{0.5,0.5,0.5,0.5,0.5,0.5}}]. \end{array}$$

For each country, we calculate the cosine distance from these hypothetical perfect scenarios. We chose the cosine distance, because the intensity of the vector is not important: what matters is its direction. We assign the country to the closest template, i.e. the one scoring the lowest cosine distance among the three. The average leftover cosine distance (energy) is a measure of how good the clustering was, i.e. how similar each country is to its assigned template.

If an exporter has a high values for the out-degree roles and low ones for in-degree roles, then it is assigned to the “Out-competing” cluster. Vice versa, low values for the out-degree roles and high ones for in-degree roles will place the country in the “Displaced” cluster. In all other cases, when the out- and in-degree roles have comparable values, the exporter is classified as “Transitioning”.

## Results

### Competition network statistical analysis

The fundamental assumption of this paper is that the competition network that we build using the methodology discussed in the previous section contains information that will allow us to predict an exporter’s future performance in the global market. If a country can out-compete many other countries in a product, then it is expected to export more of that product. The first question one might ask is: why do we need to calculate node roles? The number of times an exporter out-competes its rivals is simply its out-degree. Could this simpler statistical property inform us about export dynamics?

There are two reasons why this is not the case. The first reason is that out- and in-degree in the competition network are highly correlated. The second reason is that the competition network’s structure is more complex than one would assume.

**Fig 3:**
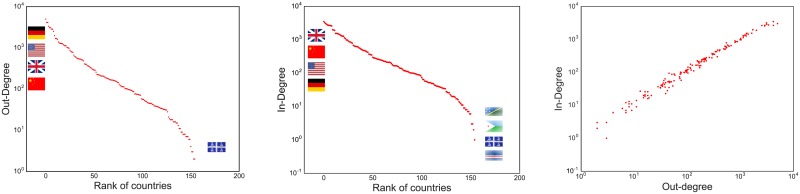
Out- and in-degree distributions. (Left) Countries are sorted and ranked in the x-axis according to their out-degree, i.e. the number of times they out-compete another country—reported in the y-axis. (Middle) The same plot, replacing the out-degree measure with the in-degree one, i.e. the number of times the country was displaced from a niche. (Right) The relationship between out-degree (x-axis) and in-degree (y-axis). Each observation is a country.

Fig 3 shows the out- and in-degree correlation. On the left we show the out-degree distribution per country, and in the middle the in-degree distribution. We can see that both distributions are very similar. In fact, the top and the bottom countries in these distributions are almost the same, sometimes in a slightly different order. On the right, we show the correlation directly. It is not possible, from this picture, to characterize any country as predominantly out-competing its rivals, because the same country will have an almost equal amount of cases in which it is displaced.

Regarding the second reason, we observe a number of topological properties that we would not expect to find in a competition network. The first one is reciprocity. When country *a* displaces country *b* in a niche, we would expect it to do so because fitter for that particular market. Yet, we observe a large number of reciprocal edges. This means that, after some time, country *b* reappears in the niche and displaces country *a*. Across our problem space (for all decades, products and parameter choice) the median reciprocity was 11.38%.

**Fig 4:**
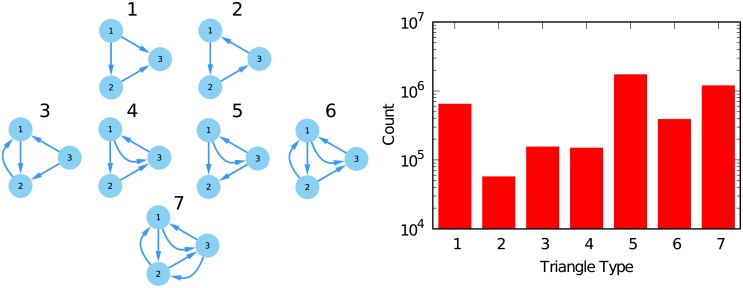
(Left) All possible triangles in a directed graph. (Right) Frequency of different types of directed triangles in the multilayer network.

The second surprising topological feature is the presence of a high number of triangles. Triangles are surprising because we would not expect a displaced country to displace a displacer. Yet, this happens frequently. Fig 4 shows on the left the seven possible types of triangles that can appear in a directed network. On the right, it depicts the counts of each type of triangle in ∼100 randomly chosen networks across all decades, products and parameter choices. Triangle types 5 and 7 are the most common, 7 being the case in which all three exporters are displacer of each other.

**Fig 5:**
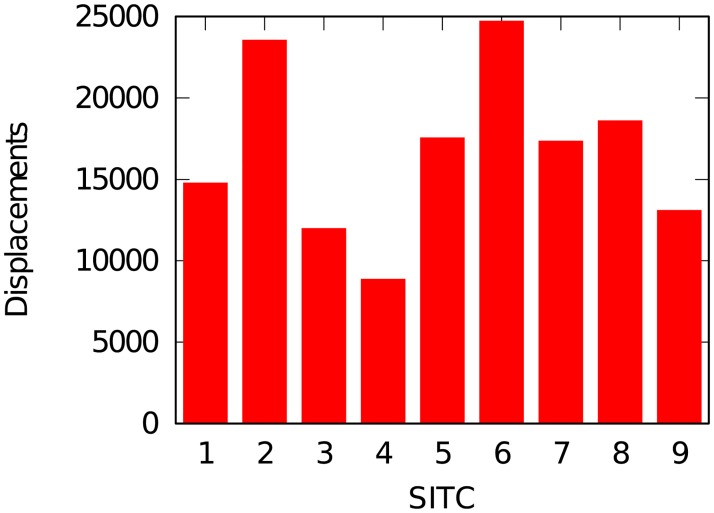
Distribution of number of displacements per one digit SITC product.

Fig 5 shows the distribution of number of displacements per one digit SITC product. We can see that there are products that are more dynamic than others.

### Role clusters

Before performing the clustering and the prediction task, we need to determine the optimal parameter choice, and evaluate the robustness of our results to this choice. Many topological properties of the multilayer networks are dependent on our choice of parameters. We investigate the direct effect on clustering quality of the three parameters *δ*, *κ*, λ. For each combination of parameter we calculate the average cosine distance between a country and the cluster template to which it is the most similar.

**Fig 6:**
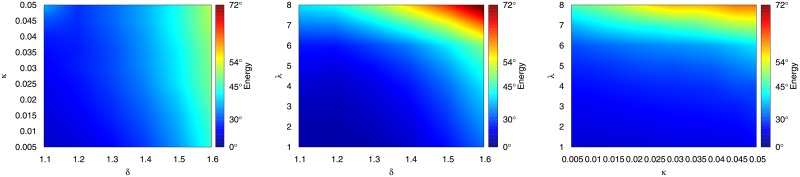
The energy landscape of the parameter space (projected over the omitted dimension). From left to right: *δ*-*κ* (λ omitted); *δ*-λ (*κ* omitted); *κ*-λ (*δ* omitted).

Since we have three parameters, the space of this search is three dimensional. To explore it, we project it into three two dimensional slices. We fix two parameters and then we calculate the average cosine distance (energy) across the omitted dimensions. Fig 6 reports the result.

From the figure, we can see that the most important parameter that creates a rugged landscape is λ—the length of a displaced exporter disappearance necessary to determine whether it is really out of the market. This is intuitive: since we are considering a decade-long period, if we require long disappearances (e.g. 8 years) the interval in which the displacement could happen becomes very narrow (e.g. only the first two years of the period). As a consequence, there are going to be very few edges in our competition network, and displacements happening after (10 − λ) years from the beginning of the decade are going to be ignored.

On the other hand, the *δ*-*κ* space is very smooth, showing that results are going to be consistent no matter the level of correlation distance we require (*δ*) or the disappearance threshold (*κ*). Between the two, *δ* seems to be more important (there is a weak left to right gradient). Again, this is unsurprising for the same reason as before: the higher the *δ* the more demanding we are in our edge creation process. For *δ* > 1.5 we start having degenerate networks which are sparser and sparser, and where triangles are impossible.

**Fig 7:**
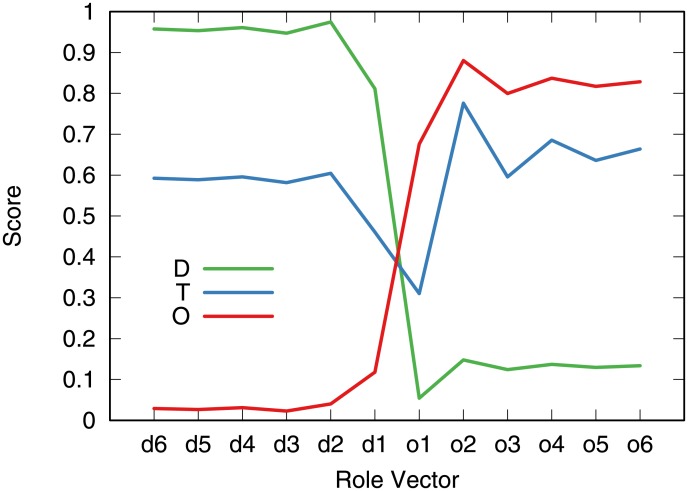
The average role-feature scores per cluster in our example. O = Out-competing, D = Displaced, T = Transitioning.

Once we fix *δ*, *κ*, λ such as to minimize the clustering energy, we obtain our final clusters, dividing countries in out-competing, transitioning and displaced for each decade and product category. As discussed in the methods section, we have three templates and countries are matched to the template most similar to them. Here, we visualize one instance of such clustering. We average the role scores for all countries in each cluster. Fig 7 depicts the result.

From the figure, we can see that the clustering procedure is able to capture the essence of the network roles. Countries in the out-competing cluster have small displaced role scores on average, and high out-competing scores. The converse is true for countries in the displaced cluster. As for the transitioning countries, they tend to have high scores in both role classes. The only exception is their low score in the first displaced role. This means that transitioning countries tend to have low in-degree, although that in-degree is generated from countries with a very high in-degree—otherwise also the other displaced scores would be low.

### Prediction

Once we fix *δ*, *κ*, λ such that we obtain the lowest residual energy (i.e. average cosine distance), we can perform a simple predictive task. We calculate the clusters using exclusively data from a given decade, say 1971 to 1980. Then, we look at the exports of each country in that product in the next decade—from 1981 to 1990. We calculate the slope of the decade trend, normalized with the maximum export value of the top exporter in that product in that period. In this way, we have for each country its competition network cluster for a decade and its corresponding export growth in the following decade. We then calculate the mean export growth rate for each country cluster. We also calculate the standard error of the mean. This is an out-of-sample prediction, since there is no information that is used both for calculating the clusters and the growth rate: the sets of years considered are disjoint.

We perform this operation for all decades for all product classes. Table 1 reports the results—S1 Table in the Supplementary Information contains the legend for each product code. Let us consider decade 1960-1970 in product 4 (fourth row). The row tells us that the countries in the out-competing cluster grew on average 4.3% per year and the ones in the transitioning cluster by.1% per year. Since the displaced cluster’s growth average was less than two standard errors from zero, we cannot be sure that their observed growth rate is significantly different from zero. The transitioning cluster was at least 2 standard errors away from zero (i.e. there is a 1 in 22 chance that the result could be observed if the null hypothesis is true); while the out-competing estimate is more than 3 standard errors from zero (1 in 370 chance of observing such result from the null hypothesis).

**Table 1:** The mean export growths per country. For each decade and product class (first two columns) we test if the corresponding clusters have an export value growth in the following decade in the same product significantly higher than zero. From left to right the means of: out-competing, transitioning, and displaced clusters. Last column is the *R*^2^ of a regression using the clusters as fixed effects. (*** 3*σ*, ** 2.5*σ*, * 2*σ*).

Decade	SITC	Out-competing	Transitioning	Displaced	*R*^2^
1960-1970	1	0.048***	0.005**	0.004**	0.151
1960-1970	2	0.039***	0.005**	0.004***	0.169
1960-1970	3	0.043***	0.005*	0.004*	0.167
1960-1970	4	0.043***	0.001*	0.003	0.129
1960-1970	5	0.144***	0.017***	0.006***	0.266
1960-1970	6	0.074***	-0.001*	0.006*	0.298
1960-1970	7	0.094***	0.007***	0.002***	0.381
1960-1970	8	0.068***	0.012**	0.008**	0.186
1970-1980	1	0.025***	0.005*	0.001**	0.165
1970-1980	2	0.024***	0.008**	0.001***	0.310
1970-1980	3	-0.019***	-0.000	-0.000	0.068
1970-1980	4	0.027***	-0.000*	0.001*	0.170
1970-1980	5	0.131***	0.002***	0.007***	0.347
1970-1980	6	0.052***	0.004***	0.003***	0.325
1970-1980	7	0.063***	0.007***	0.002***	0.401
1970-1980	8	0.107***	0.012***	0.004***	0.515
1980-1990	1	0.021***	0.002***	0.002***	0.260
1980-1990	2	0.009***	0.002*	0.001**	0.163
1980-1990	3	0.008***	-0.000	0.002	0.075
1980-1990	4	0.046***	n/a	0.002***	0.325
1980-1990	5	0.076***	0.014***	0.005***	0.305
1980-1990	6	0.027***	0.011	0.002**	0.301
1980-1990	7	0.061***	0.004***	0.002***	0.464
1980-1990	8	0.029***	0.002**	0.002**	0.148
1990-2000	1	0.042***	0.008*	0.005*	0.161
1990-2000	2	0.024***	0.004***	0.001***	0.279
1990-2000	3	0.026***	0.005*	0.003*	0.180
1990-2000	4	0.055***	0.002***	0.002***	0.183
1990-2000	5	0.103***	0.003***	0.005***	0.330
1990-2000	6	0.077***	0.007***	0.005***	0.282
1990-2000	7	0.055***	0.013*	0.001**	0.227
1990-2000	8	0.024***	0.003	0.001*	0.141
2000-2010	1	0.026***	0.006	0.005	0.063
2000-2010	2	-0.013***	-0.000*	-0.000*	0.270
2000-2010	3	0.003	-0.000	-0.002	0.005
2000-2010	4	-0.027***	0.001	0.000	0.050
2000-2010	5	0.013	0.004	-0.001	0.025
2000-2010	6	-0.015*	-0.002	-0.003	0.017
2000-2010	7	0.014	0.003	0.000	0.018
2000-2010	8	0.015**	0.001	0.001	0.037

Almost all cases considered show that countries in the out-competing clusters performed well, given that their average slope is significantly higher than zero (which would imply no growth). Both the displaced and the transitioning countries have a slope significantly lower than the countries in the out-competing cluster. In many cases they still experienced export growth, but that export growth was significantly lower than the one experienced by the out-competing countries.

There are two main deviations from this rule. The first involves product 3, which shows negative coefficients and/or lower *R*^2^. This is unsurprising, given that SITC category 3 is dominated by the product with the highest trade traffic: crude oil. Since its dynamics are more related to geological discoveries than to the ability of countries to compete, it is expected to show counter-intuitive patterns. The second exception is for all estimates using the 2000-2010 data for calculating the clusters. Also in this case this failure can be attributed to external causes. The trade data we have runs only until 2013. 2011-2013 is too short of a period to detect reliable trends, thus the test data is not good enough to evaluate our clustering.

The method works at different levels of data granularity. To test this, we repeat the full analysis, collapsing the one-digit product categories to a single product, which stands for the entire export basket of a country. Once we perform the analysis, we still find that the out-competing countries grow their exports significantly more than the transitioning and displaced countries. Table 2 reports the coefficients, per decade. For instance, in the 1970-1980 decade, the countries which were classified as out-competing in 1960-1970 grew on average almost 8%. The transitioning countries grew 1.4%, while the displaced countries grew only 0.7%. Just as in the previous case, we fail to predict the last decade for lack of long enough data.

**Table 2:** The mean export growths per country, aggregated to the total export of the country. The coefficients can be interpreted as discussed in the caption of Table 1. (*** 3*σ*, ** 2.5*σ*, * 2*σ*).

Decade	Out-competing	Transitioning	Displaced	*R*^2^
1960-1970	0.079***	0.014***	0.007***	0.269
1970-1980	0.045***	0.001**	0.004*	0.187
1980-1990	0.030***	0.000*	0.001*	0.300
1990-2000	0.058***	0.028*	0.006***	0.230
2000-2010	0.010	0.006	-0.001	0.018

**Fig 8:**
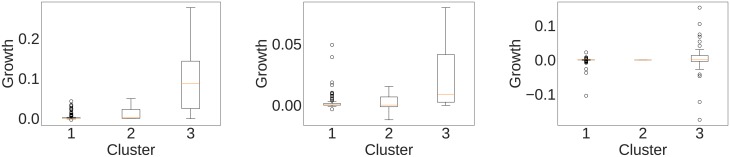
The distribution of growth rates for countries classified in the different clusters. Box plots report the 10th, 25th, 50th, 75th, and 90th percentile. Outliers are reported with circles. From left to right: 1981-90 growth in SITC product 8 (Miscellaneous manufacturing); 1991-2000 growth in SITC product 1 (Beverages and tobacco); and 2011-13 growth in SITC product 3 (Mineral fuels, lubricants and related materials).

We pick some interesting cases to represent graphically: our best, most average and worst prediction among the ones reported in Table 1. Fig 8 depicts the slope distribution in each cluster as box plots.

In our best case, the out-competing cluster was able to correctly capture all the eleven fastest growing countries in the manufacturing sector in the 80s. The twelfth country, Thailand, had less than a third the average export growth rate in the sector (∼4.92%) than the average of the top countries.

To give a better sense of this data we focus on one case from this example. Product 8 includes all manufactoring sectors, except machines (which is product 7) or manufactory chiefly focused on a single material (product 6). This category includes many products with very related machine-intensive production process, for instance a variety of garments. One of the rising economies in this sector in the 80s was China. China grew across the board in this sector, and displaced many countries in many markets. For instance, in 1986, China provided only.64% of watches imported in the United States, while France provided 1.1% (http://atlas.media.mit.edu/en/visualize/tree_map/sitc/import/usa/show/8851/1986/). By the end of the decade, in 1990 China rose almost tenfold in the market to provide 5.4% of US imported watches, while France halved to.58% (http://atlas.media.mit.edu/en/visualize/tree_map/sitc/import/usa/show/8851/1990/).

For the average case, we focus on the nine fastest growing countries, of which the out-performing cluster captured seven. The out-performing cluster captured all four countries that had an average yearly growth rate higher than 5%. Finally, the last plot shows a case in which the clustering did not manage to make sense of the export patterns. This is due to the fact that every country is an outlier in this product category, due to the importance of oil. The discovery of a large reservoir or the drying up of another one is unpredictable using the past trade patterns, and so we expect our methodology to fail in this case.

One could argue that we are capturing a random fluctuation in world trade trends. A displacement event might be a fluke of a country entering into a market niche and then exiting after some time. If this objection would be true, we should expect to observe reversion to the mean. In other words, if we use 1960 clusters to predict 1970 trade shares, then 1980 trade shares are expected to shrink by the same amount they grew in 1970. This is not the case.

**Fig 9:**
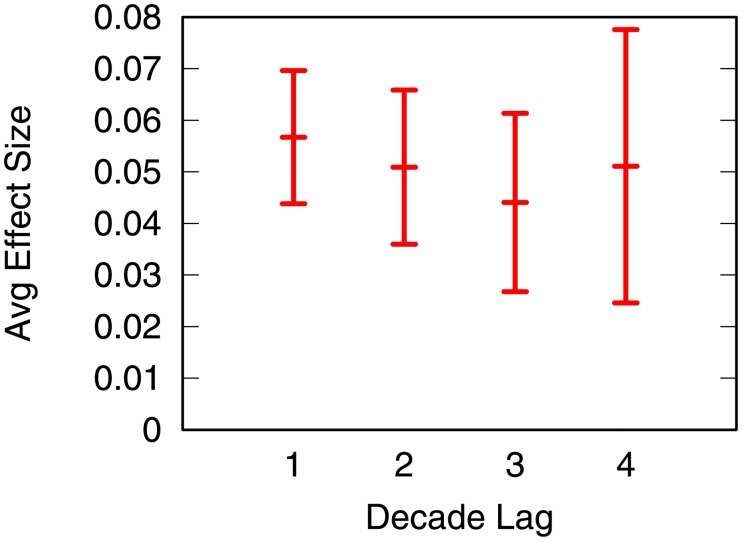
The aggregate coefficient values across all product across all decades (y axis) for increasing decade lag (x axis). A decade lag equaling one means that we predict the decade after the data used to calculate the cluster (i.e. the main result of the paper). A decade lag equaling two means we predict two decades after the cluster data: if we had cluster data from 1960 we predict 1980 growth; if we had cluster data from 1980, we predict 2000 growth.

Fig 9 shows the aggregate coefficient values across all products across all decades for increasing decade lag. In the figure, we exclude product 3 and clusters from 2000, for the reason explained above. The figure shows average and standard error of the regression coefficients, per decade lag. For instance, the first distribution (marked 1) is the average and standard error of the “Out-competing” column of Table 1. The second distribution reports the same for the regression coefficients predicting growth rates two decades away: for instance, we calculate the clusters using the 1960-1970 data and we predict the growth rate in the 1980-1990 period, i.e. two decades away. We see no sign of mean reversion. In fact, clusters from 1960 still predict—on average—a significant increase in market share in 2000, four decades later. The standard error range increase, as expected: the further away the prediction, the more uncertainty there is.

### Validation

Here we validate the role detection methodology against a series of possible objections. The first issue we address is the arbitrariness of the role detection parameters.

In the paper we delineate a procedure to choose the *δ*, *κ*, and λ parameters. The role detection strategy introduces other parameters that influence the result, such as *k*_*max*_ and *α*. However, we do not provide an equivalent procedure to choose them. The reason to fix *k*_*max*_ = 6 and *alpha* = 1 comes from their meaning. *k*_*max*_ should be set equal to the network’s diameter, because paths longer than the diameter do not provide any additional topological information. On the other hand, *α* = 1 is the most reasonable choice because it gives each role an equal weight: choosing a different weight for different role would require a reason which we cannot provide.

**Fig 10:**
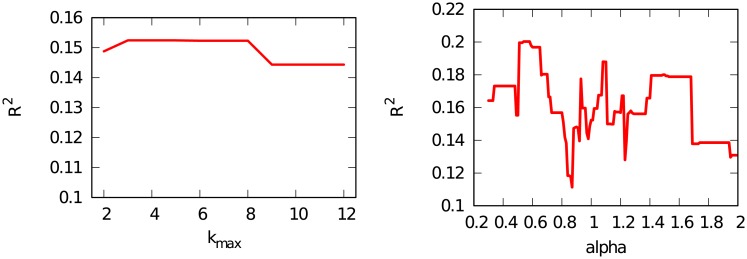
Robustness tests for *k*_*max*_ (left) and *α* (right). For different choices of these parameters, we report the effect in the *R*^2^ of the prediction. We focus on product 1 in the 1960-1970 decade.

What is the impact of these choices on the quality of our prediction? We pick product 1 in the 1960 decade to perform such exploration. Fig 10 shows their effect on the *R*^2^ of our prediction. Note that, since this test involves directly our predictive task, it cannot be used to find the optimal parameter choices, because that would imply overfitting. If our best prediction comes with, say, *k*_*max*_ = 4 we cannot set *k*_*max*_ to that value, because there would be no way to know this before running the test.

Fig 10 (left) shows that *k*_*max*_ has a minimal impact on the prediction quality. Any value between 3 and 8 is acceptable. Performance deteriorates for high values, as more and more noisy information from long paths is included, while it also deteriorates for small values, when not enough information from the network is included.

Fig 10 (right) shows that the impact of *α* is more difficult to interpret. As a result, there is no specific guidance whether to choose *α* < 1 or *α* > 1.

We now move to addressing the issue that our methodology is a correlative analysis. Correlations arise randomly even for null phenomena, provided there are enough of them. If we generate hundreds of random countries with random export patterns, some of them will have anti-correlations strong enough to clear our *δ* threshold.

To address this concern we pick 100 random triplets of exporter-importer-product. For each exporter we generate an expected export value using a zero-inflated Poisson negative binomial model—meaning that the export value is directly proportional to the total amount it exported of that product, and inversely proportional to the importer-exporter geographical distance, controlling for the fact that trade data is sparse and with a heavy tail distribution, as suggested in [33]. Then we apply our methodology to detect displacements. The expectation is that if our methodology is capturing some real phenomenon, then it should detect more displacements from the observed data than from the random data. This expectation is confirmed, since on average we observe two times more displacements than random expectation.

Still, this means that we expect half of inferred displacements to be noise. This is related to our second validation analysis. Noise connections link countries at random. In such networks, there are no non-local phenomena. Our role detection strategy operates under the assumption that the competition network is non-random, and that the *k*th role score is meaningful. If a random network with the same in- and out-degree distribution—but without any non-local phenomena—would return comparable *k*th role scores, then it means that the competition network could be dominated by the noisy connections.

To address this issue we generated 80 random networks which preserve the exact in- and out-degree distributions. Each random network is generated by picking pairs of edges at random and changing their endpoints, following [34]. We perform our analysis and we obtain the out-competing, transitioning and displaced clusters for our shuffled networks. We then calculate the adjusted mutual information between the shuffled network clusters and the observed ones. The average adjusted mutual information we obtained is equal to.1±.02 (the theoretical maximum for identical clusters is 1, and 0 means completely independent clusters). We consider this as an argument supporting our clustering, given that shuffled networks with no non-local interactions return clusters which are not related with the ones we observe.

Moreover, the clusters obtained from the shuffled networks do not divide countries well when it comes to their export growth. We replicate the result for the 1960-1970 decade in product 1 (first row of Table 1). The clusters from the shuffled network returned very similar growth rates with each other, and significantly different from the non-shuffled network ones: 1.47% (shuffled) vs 4.8% (observed) for out-competing, 1.45% (shuffled) vs 0.5% (observed) for transitioning, and 1.24% (shuffled) vs 0.4% (observed) for displaced. The shuffled network preserved the in- and out-degrees but disrupted non-local dynamics, and this analysis proved that this disruption significantly affects the ability of sorting through the countries.

A third robustness check involves our clustering procedure. Since we compare the exporter role vectors to templates, our clustering is supervised, i.e. we impose what the clusters should look like. On the one hand, this enhances the interpretability of the extracted clusters, on the other hand it might introduce biases. We test for possible biases by designing an unsupervised version of the clustering.

In this version, we still fix the number of desired clusters to three (out-competing, transitioning, displaced), but we do no provide templates. Rather, we run a kMeans algorithm on the role matrix. We then correlate the results of the supervised and unsupervised clustering. We perform this test on a subset of our parameter space. We obtain a correlation of.932 ± .032. Since we obtain a very high correlation, we conclude that using a supervised strategy did not introduce significant bias: the extracted clusters are virtually indistinguishable from the ones extracted with an unsupervised technique.

**Fig 11:**
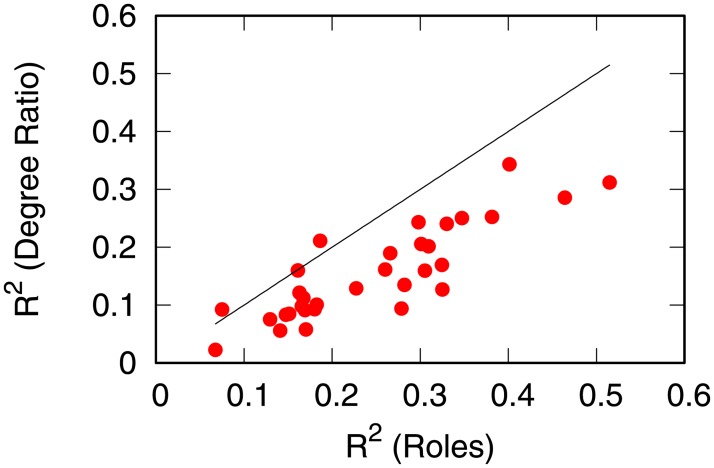
The relationship between the *R*^2^ export growth prediction using the role clusters (x-axis) and using the logarithm outdegree/indegree ratio (y-axis). Each observation is a decade-product combination: the x-axis values are the *R*^2^ values reported in Table 1, excluding product 3 and the 2000-2010 decade. The black line is the identity line: observations below the line are the ones for which the role clusters performed better than the log degree ratio.

Finally, we test whether the role detection and the clustering procedure are necessary at all. When motivating the method we use, we showed that the outdegree and the indegree are highly correlated, thus they cannot be used for prediction. However, one could use their difference for making the prediction. In Fig 11 we show the predictive power such operation has. We predict the growth in export with the logarithm of the outdegree/indegree ratio. In all cases but two, such test returns worse results than the role detection method—shown in the figure below the identity line.

Moreover, by clustering the role scores we are compressing their information: we go from a vector of 12 numbers to a single variable that can have only three values (out-competing, transitioning, displaced). We do so because we believe that the role vectors might have fluctuations that might introduce noise, and that noise will cancel out if we cluster the vectors. To verify if this is the case, we test the same linear regression model we used in the previous section, using the 12 role scores instead of the cluster labels. Every single model has lower *R*^2^ than the corresponding model using the cluster labels (average −.073 ± .044). We can conclude that the clusters are indeed improving the quality of the prediction.

## Discussion

In this paper, we adopted an ecosystem approach to the analysis of the global trade patterns. We see exporters as organisms competing for resources in different market niches. A market niche is a country importing a product. The assumption is that exporters want to out-compete other exporters, attempting to occupy the entire market niche. The appearance of a new exporter in a niche can be followed by the disappearance of another country. This is what we call a displacement event. We create a formal definition of displacements and we systematically collect all of them along a period spanning fifty years. A displacement event can be represented as a directed edge going from the out-competing exporter to the displaced one. We call the collection of all displacements a “competition network”, which is a weighted directed multilayer network, where each layer is a product class.

While the in- and out-degree of a node in a competition network have an intuitive interpretation—being the number of displacements experienced and caused by an exporter, respectively –, we show that in practice these measures cannot be used for classifying countries. The reason is their very high correlation. To fix this issue, we calculate network roles based on in- and out-degree flows. By clustering nodes according to their role score, we are able to classify them in three categories: out-competing, transitioning, and displaced. We show that these classes can be used to predict the future performance of an exporter in a particular market, in term of growth of total export value.

Our methodology has several issues. First, it does not consider actual displacements: the edge creation process is correlative by design, so we are not really capturing if the appearance of a new exporter really caused the disappearance of another. Second, it cannot be applied to all product classes: our predictions fail when considering natural resources composing the vast majority of some countries’ exports, such as crude oil. Finally, we have not built a formal theory of why the competition network roles are predictive: we do not control for confounding factors that might drive both growth in exports and the position of a country in the network.

## Conclusion

Notwithstanding the issues discussed in the previous section, our paper provides a useful tool to make sense of the current export patterns, and it paves the way for future research. The fact that we cannot predict the growth in natural resources is not crucial, as it makes little sense to plan a development strategy by aiming at discovering oil. Countries are more interested in developing capabilities for sustainable growth. More importantly, even if we cannot disentangle roles from other confounding factors, our methodology can be used as an easy-to-implement canary indicator to identify future market-leading exporters in a given product. Given that success in exporting a product can be a telltale sign of other societal indicators such as income inequality [35] and poverty traps [36], the reach of our methodology can span multiple potential applications.

We see several future developments for this paper. First, we performed our analysis at a very aggregated product classification level (one digit SITC codes). We can increase the level of detail up to four digits (from ten to a thousand products). At such granularities, new challenges arise: displacements in a product might be predictive of growth in other, more profitable products, as countries might move from textile to machinery manufacturing. Second, we could tackle the issue of causality, investigating case studies of actual displacements that took place in economics history. Finally, we could explore the confounding factors of our predictive task, and identify which factors—relevant for economics thinking—are determining the position of exporters in the competition network.

## Supporting information

S1 File The file contains the data and code to reproduce the main results in the paper, namely Table 1 and Fig 8. (ZIP)

S1 Table The SITC product classification legend, showing the correspondence between each product code and its label. (PDF)
