# Supplementary material for: The struggle for existence in the world market ecosystem
Source: PLoS One. 2018 Oct 3;13(10):e0203915. doi: 10.1371/journal.pone.0203915 (PMC6169900; doi:10.1371/journal.pone.0203915)
Supplement: S1 Table — (PDF) [file pone.0203915.s002.pdf]

Supplementary Information for: The Struggle for Existence in the World Market Ecosystem

Viviana Viña-Cervantes[1], Michele Coscia[1,2,3,*], and Renaud Lambiotte[1,4].

1 Naxys Department of Mathematics, University of Namur, Namur, Belgium
2 Center for International Development, Harvard University, Cambridge, MA, USA
3 IT University of Copenhagen, Copenhagen, DK
4 Mathematical Institute, University of Oxford, Oxford, UK

* michele_coscia@hks.harvard.edu

Appendix A: Product Classification

Code	Label
1	Beverages and tobacco
2	Crude materials except fuels
3	Mineral fuels, lubricants and related
4	Animal and vegetable oils, fat and waxes
5	Chemical and related products
6	Manufactured goods classified chiefly by material
7	Machinery and transport equipment
8	Miscellaneous manufactured articles

Table S1: The SITC product classification legend, showing the correspondence between each product code and its label.
